# Evaluation of machine learning approach for surgical results of Ahmed valve implantation in patients with glaucoma

**DOI:** 10.1186/s12886-024-03510-w

**Published:** 2024-06-11

**Authors:** Seung Yeop Lee, Dong Yun Lee, Jaehong Ahn

**Affiliations:** 1 Department of Ophthalmology, Ajou University Medical Center, Ajou University School of Medicine, 164, World Cup-ro, Yeongtong-gu, Suwon, 16499 Republic of Korea; 2 Department of Biomedical Informatics, Ajou University School of Medicine, 154, Word Cup-ro, Yeongtong-gu, Suwon, 16499 Republic of Korea

**Keywords:** Machine learning, Ahmed valve implantation, Glaucoma

## Abstract

**Background:**

Ahmed valve implantation demonstrated an increasing proportion in glaucoma surgery, but predicting the successful maintenance of target intraocular pressure remains a challenging task. This study aimed to evaluate the performance of machine learning (ML) in predicting surgical outcomes after Ahmed valve implantation and to assess potential risk factors associated with surgical failure to contribute to improving the success rate.

**Methods:**

This study used preoperative data of patients who underwent Ahmed valve implantation from 2017 to 2021 at Ajou University Hospital. These datasets included demographic and ophthalmic parameters (dataset A), systemic medical records excluding psychiatric records (dataset B), and psychiatric medications (dataset C). Logistic regression, extreme gradient boosting (XGBoost), and support vector machines were first evaluated using only dataset A. The algorithm with the best performance was selected based on the area under the receiver operating characteristics curve (AUROC). Finally, three additional prediction models were developed using the best performance algorithm, incorporating combinations of multiple datasets to predict surgical outcomes at 1 year.

**Results:**

Among 153 eyes of 133 patients, 131 (85.6%) and 22 (14.4%) eyes were categorized as the success and failure groups, respectively. The XGBoost was shown as the best-performance model with an AUROC value of 0.684, using only dataset A. The final three further prediction models were developed based on the combination of multiple datasets using the XGBoost model. All datasets combinations demonstrated the best performances in terms of AUROC (dataset A + B: 0.782; A + C: 0.773; A + B + C: 0.801). Furthermore, advancing age was a risk factor associated with a higher surgical failure incidence.

**Conclusions:**

ML provides some predictive value in predicting the outcomes of Ahmed valve implantation at 1 year. ML evaluation revealed advancing age as a common risk factor for surgical failure.

**Supplementary Information:**

The online version contains supplementary material available at 10.1186/s12886-024-03510-w.

## Background

Glaucoma is a progressive optic neuropathy and the leading cause of irreversible blindness [1]. Glaucoma development demonstrates many risk factors, but lowering intraocular pressure (IOP) is the only significant risk factor to slow glaucoma progression [2, 3]. Surgical therapy is an option when medical therapy does not achieve the target IOP. Trabeculectomy and glaucoma drainage device implantation (GDI) remain the most commonly performed surgical therapy for patients with open angle glaucoma, although interest and availability of micro-invasive glaucoma surgery procedure are recently increasing [4, 5]. Especially, the proportion of GDI is increased in favor of the growing experience of glaucoma surgeons [6–8]. The commonly used device among GDI is the Ahmed valve. The Ahmed valve allows aqueous humor to flow freely through a silicone tube, usually placed in the anterior chamber, towards a polypropylene plate. Reported success rates with Ahmed valve range between 60% and 82% at 2 years, and 49% at five years of follow-up, with a failure rate of 10% per year [9, 10].

The safety and efficacy evaluations of Ahmed valve were studied by multicenter, randomized clinical trial, which is represented as Ahmed Baerveldt Comparison Study (ABC) [11] and Ahmed Versus Baerveldt Study (AVB) [12], by increasing the interest in GDI, and a study revealed the risk factor associated with surgical failure [13]. Preoperative IOP, neovascular glaucoma (NVG) and young age were determined as significantly associated risk factors with surgical failure [13]. Moreover, a previous glaucoma surgery [9], the presence of a hypertensive phase [14], and the surgeon’s experience [15] appeared to be associated with the survival of the Ahmed valve implant. However, the limitations of these studies lie in their retrospective design, and determining the surgical progression by considering all of the risk factors is limited in the real world. Instead, predicting the patient’s surgical success rate is appropriate, using several categories of ophthalmic and non-ophthalmic factors, including systemic diseases such as hypertension and diabetes, and systemic medication, such as psychiatric medication, which is hypothesized to play a role in glaucoma development and progression [16, 17] when determining surgical plan. For this purpose, machine learning (ML) techniques have been widely used for predicting surgical success in ophthalmology. Koprowski et al. compared artificial neural networks topologies to evaluate corneal power which is related to surgical success after myopic corneal refractive surgery [18]. Liu et al. conducted ML to predict the postoperative depth of focus after cataract surgery with Tecnis Symfony implantation and evaluated associated impact factors [19]. When using ML algorithms, the inclusion of multiple variants is essential and performing comparative analysis using multi-source input combinations is imperative to improve ML performance [20]. Previous studies associated with glaucoma surgery have effectively used these aspects. Banna et al. used ML techniques (random forest, support vector machine, artificial neural network) to predict trabeculectomy surgical outcomes [21]. Paul et al. successfully identified the features that are associated with surgical failure of Molteno and Baerveldt implant using the ML technique (artificial neural network, random forest, decision tree, support vector machine) [22]. Through previous studies utilizing ML, it has become evident that ML offers significant advantages in modeling complex non-linear and conditional relationships, thereby generating individual patient-level predictions. By utilizing the ML model more accurate predictions of surgical outcomes for individual patients can be achieved. However, no studies have reported in association with Ahmed valve until now.

Therefore, this retrospective study aimed to evaluate the ability of ML models to predict the surgical outcomes of Ahmed valve implantation in patients with glaucoma using multiple combinations of available datasets, including preoperative patient’s demographics, systemic data, such as psychiatric medication, and ophthalmic parameters, as a first attempt.

## Methods

### Patients

This study entailed ML model development and evaluation based on single-center and retrospective data. We reviewed the medical records of patients with glaucoma who were unresponsive to medical treatment and had undergone Ahmed valve implantation for uncontrolled IOP from January 2017 to December 2021 at Ajou University Hospital. The ethics committee and the Institutional Review Board of Ajou University Hospital (AJOUIRB-DB-2023-069) approved this study which followed the tenets of the Declaration of Helsinki. The inclusion criteria were primary open-angle, uveitic, neovascular, pseudoexfoliation and primary angle closure glaucoma with an IOP > 21mmHg despite the maximum tolerated oral and topical antiglaucoma medical therapy, and vision better than light perception preoperatively. This study included cases with previous cataract surgery, vitrectomy, and trabeculectomy. Exclusion criteria included cases with patients who are < 18 years old and patients with < 12 months of follow-up period.

We collected preoperative data, including patient demographic data, systemic health data, such as systemic medication and disease, glaucoma diagnosis, and preoperative and postoperative ophthalmic parameters by reviewing the medical records. All of the patients received complete ophthalmic examination, including slit-lamp examination, IOP measurement, gonioscopy, and fundus examination to gain ophthalmic parameters. The Goldmann applanation tonometry was used to measure IOP, and experienced examiners performed specular microscopy (SP-2000P; Topcon Corp., Tokyo. Japan).

### Outcome measurements

Postoperative data were obtained from the records of patients from all consecutive visits. Data included IOP, the number of antiglaucoma medications, and visual acuity (best-corrected visual acuity). These data were collected at 1, 2, 3, 6, and 12 months postoperatively. Decimal visual acuity was converted to the minimum angle of resolution (logMAR), and any fixed-dose combination drugs were considered as two separate drugs [23].

The primary outcome included the surgical failure of Ahmed valve implantation at the 1-year postoperative visit. Surgical failure was defined as [1] an IOP > 21mmHg or < 5 mmHg or IOP reduced by less than 20% from the baseline at the two consecutive visits after 3 months; [2] additional glaucoma surgery due to inadequately controlled IOP; [3] loss of light perception postoperatively. Eyes that failed to achieve the above criteria were considered successful. Intervention, such as laser suture lysis or bleb needling, were considered normal postoperative care and was, therefore, not recorded as either reoperation or failure.

### Surgical procedures

**Fig. 1:**
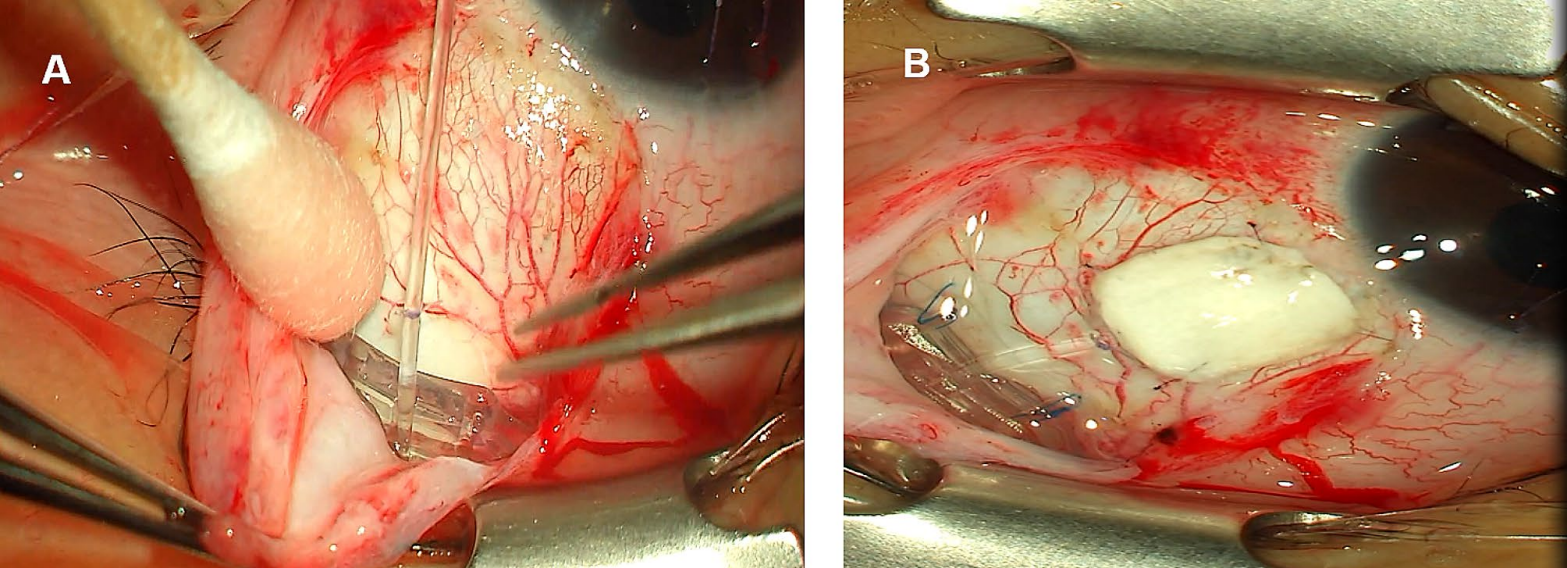
Surgical procedure of Ahmed valve implantation. The fornix-based conjunctival flap was created in the superotemporal quadrant and the Ahmed implant is firmly fixed to the scleral with 8–0 prolene sutures in all of the cases (**A**). The Ahmed tube is positioned in the anterior chamber in most of cases (**B**), except in 28 cases in which the Ahmed tube was inserted into the ciliary sulcus. A human-donor scleral graft was placed on the tube and sutured to the sclera with 10–0 nylon suture in 93 cases (**B**). The other 60 cases used a bovine pericardial patch graft for Ahmed valve implantation

A single experienced glaucoma specialist (J.A.) completed all patient surgeries using standard documented techniques and performed Ahmed valve implantation only in the superotemporal quadrant. A fornix-based conjunctival flap was created, and the bare sclera was dissected (Fig. 1). A sponge soaked with 0.04% Mitomycin was applied for 5 min to the bare sclera, followed by irrigation with balanced salt solution (BSS). Ahmed valve was primed using 26G cannula, injecting BSS, and making a partial ligation of the tube with 8–0 vicryl 6 to 8 mm posteriorly to prevent of early postoperative hypotony [24]. The plate of the Ahmed valve (model FP-7; New World Medical Inc., Rancho Cucamonga, CA, USA) was positioned 8–10 mm posterior from the limbus and sutured to the sclera with two 7–0 prolene sutures. The drainage tube was inserted into the ciliary sulcus in 28 cases and into the anterior chamber in most cases, through the needle track. It was covered with a 4 × 4 mm human-donor scleral patch in 93 cases and bovine pericardial patch graft in 60 cases that was fixed with 10–0 nylon. Finally, tenon’s capsule and the conjunctiva were closed over the Ahmed valve using 10–0 nylon. Postoperatively, all patients were treated with 0.5% levofloxacin hydrate (Cravit, Santen, Osaka, Japan) 4 times daily, 1% prednisolone acetate (Pred Forte, Allergan, Irvine, CA, USA) 4 time daily, and bromfenac sodium hydrate (Bronuck Oph Soln, Taejoon, Seoul, Korea) twice daily, all of which subsequently were gradually tapered.

### Model development

The patients were randomly categorized into training and test datasets at a ratio of 7:3 to accurately estimate predictive performance. Three-fold cross-validation using the training dataset was used in the model development due to an insufficient number of subjects for an additional validation set to optimize hyperparameters [25]. One of the three subsets was used as the validation dataset for the model and the remaining two subsets were utilized as training datasets each round, and the process continued for three rounds. This provides a more stable and reliable way to measure the model performance.

Firstly, we developed the models using three algorithms, using only ophthalmic parameters through the process described above. The algorithms included logistic regression (LR), extreme gradient boosting (XGBoost), and support vector machines (SVM) which are widely used in clinical science [26, 27]. LR model a relationship between the categorical response variable and covariates. LR performs as well as ML techniques in predicting disease risk and has the advantage of high explanatory power [28]. SVM is a popular ML technique for classification problems in many biomedical fields. SVM performs classification by constructing a multidimensional hyperplane that optimally discriminates between two classes by maximising the margin between two clusters of data [29]. XGBoost algorithm is a decision tree-based model on the training dataset. XGBoost starts with a simple initial model and its residuals and misclassifications are iteratively improved in subsequent models searching from among all available predictors to try to minimize misclassification. XGBoost was commonly chosen for its interpretability of results and robustness to overfitting [30]. We evaluated each model according to accuracy (ACC), area under the receiver operating characteristics curve (AUROC), area under the precision-recall curve (AUPRC), and F1 score to analyze predictive performance [31, 32]. The algorithm with the best performance was selected as the final algorithm according to the AUROC.

Secondly, we developed further prediction models based on the combination of data types, using the final selected algorithm. Three socio-demographic parameters, such as age or sex, 22 ophthalmic parameters, 6 systemic medical records, such as hypertension or diabetes, and 4 kinds of psychiatric medications from the hospital electronic health record were used (Table 1). We reclassified these parameters into three primary datasets to simplify the model analysis process. Dataset A consisted of the socio-demographic and ophthalmologic features, dataset B consisted of systemic disease and medications, and dataset C consisted of psychiatric medications. Among the patients in this study, there was an unusually high prevalence of those taking various types of psychiatric medication despite the unknown precise disease diagnosis. Therefore, we decided to categorize these psychiatric data into a single category as dataset C. Finally, a total of four prediction models were developed through the final algorithm according to the combination of dataset A, B, and C.

**Table 1:** Univariate analysis of demographic, ophthalmologic, systemic and psychiatric features

Characteristics	Surgical success (*n* = 131)	Surgical failure (*n* = 22)	*p*-value
**Socio-demographic features**			
Male (n, %)	91 (69.5)	16 (72.7)	0.95
Age (Mean, SD)	62.7 (14)	60.2 (13)	0.45
Race (Korean) (n, %)	131 (100.0)	22 (100.0)	1.00
**Ophthalmologic factors**			
History of previous trabeculectomy (n, %)	20 (15.3)	7 (31.8)	0.11
History of previous vitrectomy (n, %)	35 (26.7)	8 (36.4)	0.50
History of previous SLT (n, %)	28 (21.4)	6 (27.3)	0.74
History of previous LI (n, %)	7 (5.3)	0 (0.0)	0.58
History of previous cataract operation (n, %)	36 (27.5)	8 (36.4)	0.55
Anatomy of closed angle (n, %)	13 (9.9)	0 (0.0)	0.26
Number of pre-operative topical medication (Mean, SD)	3.7 (0.8)	3.6 (0.7)	1.00
Number of pre-operative PG analogue (n, %)	114 (87.0)	18 (81.8)	0.75
Number of pre-operative alpha-agonist (n, %)	118 (90.1)	18 (81.8)	0.44
Number of pre-operative topical CAI (n, %)	122 (93.1)	21 (95.5)	1.00
Number of pre-operative beta blocker (n, %)	125 (95.4)	22 (100.0)	0.67
Number of pre-operative oral CAI (n, %)	75 (57.3)	13 (59.1)	1.00
Pre-operative IOP, mmHg (Mean, SD)	29.8 (7)	30.2 (9)	0.85
Pre-operative VA, logMAR (Mean, SD)	0.7 (0.4)	0.7 (0.4)	0.84
Co-operation with cataract extraction (n, %)	15 (11.5)	3 (13.6)	1.00
Used patch (pericardium) (n, %)	53 (40.5)	7 (31.8)	0.60
Position of tube (sulcus) (n, %)	21 (16.0)	7 (31.8)	0.14
Corneal central thickness, μm (Mean, SD)	531.6 (44.8)	544.5 (33.7)	0.20
Diagnosis as primary open angle glaucoma (n, %)	46 (35.1)	8 (36.4)	1.00
Diagnosis as primary angle closure glaucoma (n, %)	13 (9.9)	0 (0.0)	0.26
Diagnosis as neovascular glaucoma (n, %)	51 (38.9)	10 (45.5)	0.73
Diagnosis as pseudoexofoliation glaucoma (n, %)	5 (3.8)	0 (0.0)	0.78
Diagnosis as uveitic glaucoma (n, %)	16 (12.2)	3 (13.6)	1.00
**Systemic conditions, n (%)**			
Hypertension	51 (38.9)	10 (45.5)	0.73
Hyperlipidemia	14 (10.7)	2 (9.1)	1.00
Diabetes	85 (64.9)	7 (31.8)	0.01
Chronic kidney disease	12 (9.2)	0 (0.0)	0.29
Myocadiac infarction	13 (9.9)	1 (4.5)	0.68
Arrhythmia	3 (2.3)	2 (9.1)	0.31
Asthma	7 (5.3)	1 (4.5)	1.00
**Systemic medications, n (%)**			
Anti-hypertensive drugs	38 (29.0)	7 (31.8)	0.99
Statin	38 (29.0)	7 (31.8)	0.99
Steroid	27 (20.6)	6 (27.3)	0.67
**Psychiatric medications, n (%)**			
Benzodiazepine	44 (33.6)	9 (40.9)	0.67
Antidepressants	11 (8.4)	1 (4.5)	0.85
Antipsychotics	6 (4.6)	0 (0.0)	0.67
Mood stabilizer	4 (3.1)	1 (4.5)	1.00

*n* = number; SD = standard deviation; SLT = selective laser trabeculoplasty; LI = laser iridotomy; PG = prostaglandin analogue; CAI = carbonic anhydrase inhibitor; IOP = intraocular pressure; VA = visual acuity; logMAR = logarithm of the minimum angle of resolution

### Statistical analysis

This study appropriately performed descriptive statistical analyses. Baseline characteristics are presented as counts with proportions for categorical variables and as mean with standard deviation for continuous variables. Baseline demographic and clinical characteristics were compared using χ2 tests and independent t-tests. ACC, AUROC, AUPRC and F1 score were calculated to evaluate the prediction model performance. We used the maximal Youden index to select the optimal cut-off value in the prediction model [33]. The three ML algorithms evaluated in this paper are LR, SVM (R package e1071), and XGBoost (R package xgboost). All *p-values of* < 0.05 were considered to represent statistical significance. R software version 4.1.0 (R Foundation for Statistical Computing, Vienna, Austria) and open-source statistical R packages were used for all statistical analyses.

## Results

### Baseline characteristics

The model development included 133 of the 272 patients who underwent Ahmed valve implantation within the designed period based on our exclusion criteria. The 139 patients excluded from the study either did not undergo follow-up observation for 1 year after surgery or had persistently measured visual acuity at hand motion or below after surgery. This study included 20 with bilateral involvement out of 133 patients. Consequently, a total of 153 eyes were evaluated at postoperative 1 year. The surgical success rate was 85.6%, 131 eyes were categorized as the surgical success group, and 22 as the surgical failure group. Fourteen eyes in the surgical failure group had elevated IOP, while three eyes underwent reoperation and five eyes underwent visual loss after primary surgery.

A total of 38 preoperative parameters were collected for prediction modeling, including 3 socio-demographic features, 22 ophthalmologic features, 6 comorbid conditions, 3 systemic medications and 4 psychiatric medications. Table 1 shows the results of each parameter in the surgical success and failure groups. Demographic and ophthalmologic datasets and psychiatric datasets demonstrated no significant difference between the two groups. The systemic feature of diabetes is the only significantly different factor between the two groups (*p* = 0.01).

### Prediction models

A total of 108 individuals were selected as the training group and 45 individuals as the test group based on a random split of the total patients into training and test datasets at a ratio of 7:3. Among the randomly divided 108 individuals of the training group, 15 were the patients who had successfully controlled IOP after Ahmed valve implantation, while among the 45 patients in the test group, 7 patients had well controlled IOP after surgery.

**Fig. 2:**
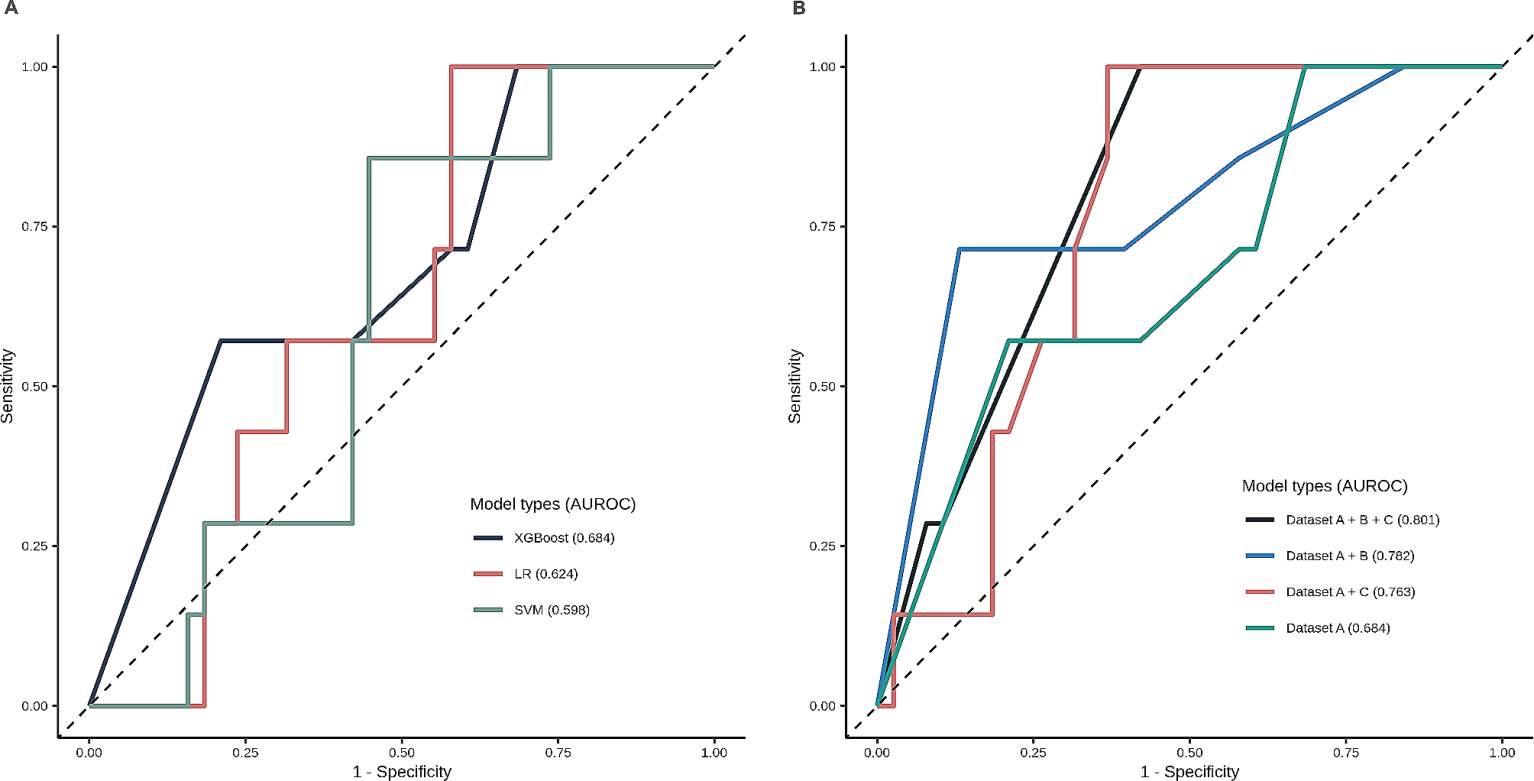
Receiver operating characteristic (ROC) curve of the model predicting the surgical failure of Ahmed valve implantation. (**A**) ROC curve for the models according to algorithms for demographic and ophthalmologic features. (**B**) ROC curve for the XGBoost model trained on four types of dataset combinations. Dataset A indicates demographic and ophthalmologic factors, dataset B represents systemic factors, and dataset C denotes psychiatric factors. The performance of the models using the area under the ROC curve is compared

The performance evaluation of the ML model in the demographic and ophthalmologic datasets (dataset A) included SVM, LR, and XGBoost to select the final algorithm (Fig. 2A). The comparison of the average AUROC values revealed XGBoost to demonstrate the best-performance model in dataset A, achieving a score of 0.684. XGBoost also provided the highest accuracy of 0.756 compared to the accuracy of 0.600 in SVM and 0.511 in LR from dataset A. Table 2 shows the performance metrics of three predictive models from dataset A. The final model of XGBoost was evaluated using the expanded datasets, dataset B and C. The models with two or more datasets in the expanded datasets outperformed those using only dataset A in terms of AUROC (Table 2). Furthemore, the XGBoost model for dataset A, B, and C showed the highest AUROC (Fig. 2B, A + B: 0.782; A + C: 0.773; A + B + C: 0.801). However, the model using datasets A and B performed better than the other models based on accuracy and F1-score (an accuracy of 0.844 and an F1 score of 0.588, Table 2). The results of evaluating the expended dataset using SVM and LR are shown in the Supplementary Table 1 [see Additional file 1].

**Table 2:** Performance results of predictions models

Datasets	A			A + B	A + C	A + B + C
Performance metrics	SVM	Logistic regression	XGBoost	XGBoost	XGBoost	XGBoost
ACC	0.600	0.511	0.756	0.844	0.689	0.644
AUROC	0.598	0.624	0.684	0.782	0.763	0.801
AUPRC	0.172	0.184	0.108	0.095	0.100	0.095
F1 score	0.400	0.389	0.421	0.588	0.500	0.467
Sensitivity	0.857	1.000	0.571	0.714	1.000	1.000
Specificity	0.553	0.421	0.789	0.868	0.632	0.579
Precision	0.261	0.241	0.333	0.500	0.333	0.304

Dataset A indicates demographic and ophthalmologic factors, dataset B represents systemic factors, and dataset C denotes psychiatric factors. ACC = accuracy; AUROC = area under the receiver operating characteristics curve; AUPRC = area under the precision recall curve; SVM = support vector machine

### Predictive features of models

**Fig. 3:**
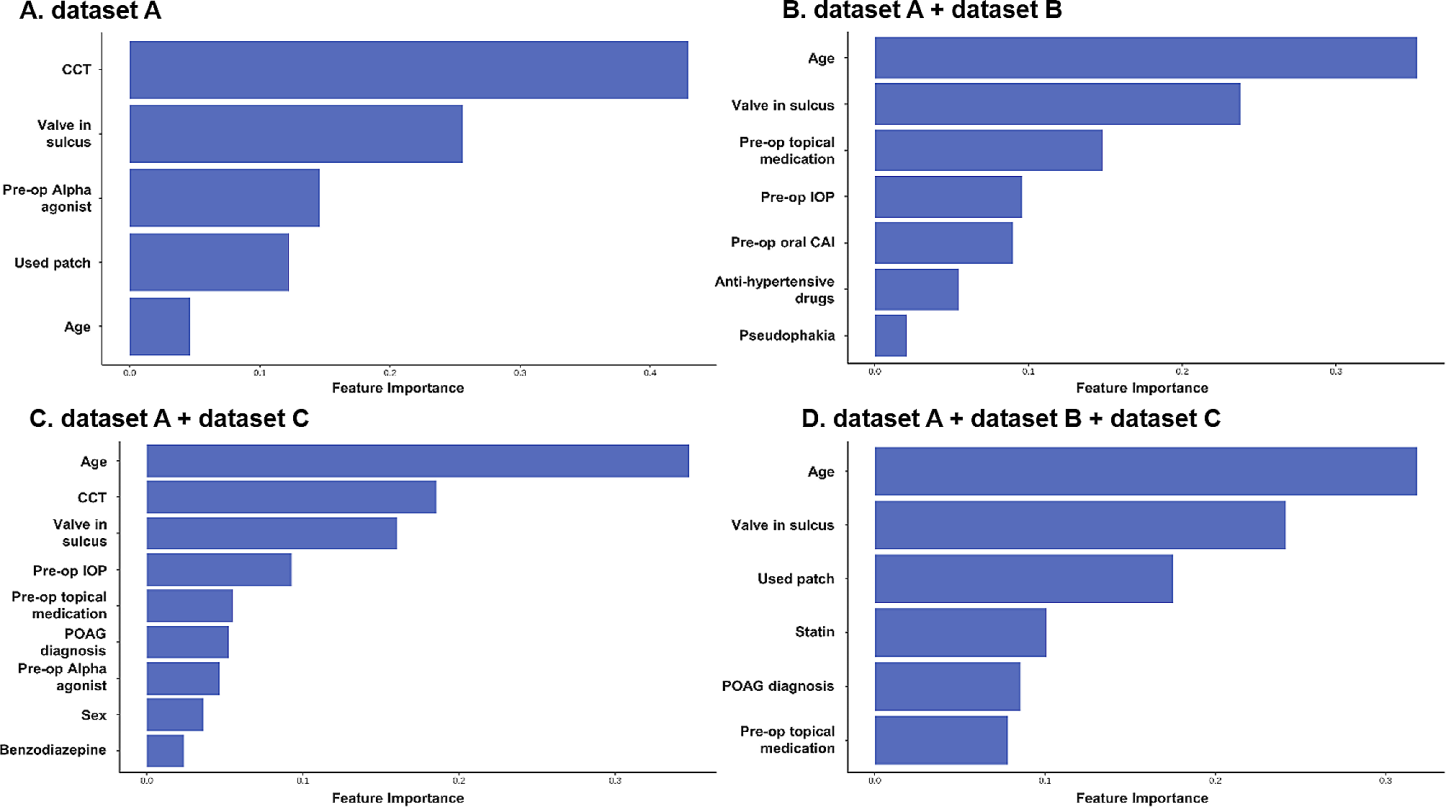
The important predictive features of the XGBoost model from the four types of dataset combinations. Dataset A indicates demographic and ophthalmologic factors, dataset B represents systemic factors, and dataset C denotes psychiatric factors

Figure 3 shows the important predictive features of the XGBoost model from dataset A, B, and C. The Supplementary Table 2 shows the positive or negative contributors to the surgical failure prediction using XGBoost in more detail [see Additional file 1]. According to dataset A, the corneal central thickness appears as the most significant feature, indicating an increased risk of surgical failure associated with thinner cornea (Fig. 3A). The combination of dataset B and C demonstrated age as the most important feature, indicating that advancing age is a risk factor associated with a higher surgical failure incidence (Fig. 3B and C). The XGBoost model applied to dataset A, B, and C revealed that age remains the most crucial feature associated with an elevated risk of surgical failure (Fig. 3D). Additionally, the position of the valve appears as the next important feature, indicating that the sulcus positioning of the Ahmed valve is associated with a higher surgical failure incidence.

## Discussion

This study developed a model to predict the occurrence of surgical failure in Ahmed valve implantation 1 year postoperatively using ML algorithms. This is the first attempt to apply ML algorithms for predicting the outcome of Ahmed valve implantation. This retrospective study investigated as many factors as possible from preoperative patient data in the medical records of Ajou university hospital to compare the different ML models. The factors included not only ophthalmologic factors, but also those related to systemic diseases, such as hypertension, diabetes and psychiatric medications, based on increasing evidence about the role of systemic conditions and medication in glaucoma pathophysiology [34]. Consequently, the XGBoost model demonstrated the highest performance based on AUROC in this study, and the high performance of our models confirms that the ML algorithms using preoperative patient data would enable clinicians to provide tailored treatment options, such as selecting a surgical method expected to yield a higher success rate. Additionally, age is determined as the strongest factor associated with surgical failure.

Few studies have investigated the surgical success rate of Ahmed valve implantation and the risk factor associated with surgical failure. Bowden et al. revealed that lower preoperative IOP, NVG, and younger age are associated with surgical failure predictors [13]. The study used pooled data from the ABC, AVB, and the Tube Versus Trabeculectomy study and revealed the 38.3% cumulative probability of surgical failure of the overall study group at 5 years. Souza et al. also revealed a cumulative probability of 80% and 49% at 1 year and 5 years, respectively. They found that previous history of glaucoma surgery and the silicone type of Ahmed valve (FP-7) were associated with an increased risk of surgical failure [35]. Djodeyre et al. demonstrated a cumulative surgical success rate of 70.1% and 63.7% at 12 and 24 months, respectively, and revealed both congenital glaucoma and surgical experience as risk factors associated with surgical failure, although the study focused on pediatric glaucoma [15]. Most of the identified risk factors in these studies are difficult to modify or influence preoperatively. Based on the limitation of previous studies, our study aimed to find and include modifiable risk factors associated with surgical failure by approaching ML algorithms. These factors include the type of systemic medication or topical medication used preoperatively, as well as the type of patch used during surgery and the positioning of the Ahmed valve. We incorporated as many variable preoperative factors as possible, including socio-demographic, ophthalmologic, and systemic factors, to enhance predictive value and thus accomplish our purpose. Specifically, we included psychiatric medications that have been increasingly used concomitantly for patients with glaucoma in recent times in terms of systemic factors [16].

Three models using a combination of datasets showed similar tendencies for the top two important features with the XGBoost model in terms of feature importance. Age is the most important predictive feature, regardless of dataset combinations. Advanced age was identified as a significant risk factor associated with increased surgical failure. Our result is contrasted with the previous study revealing that younger age is associated with an increased risk of surgical failure [13]. The discrepancies between the two studies are attributed to differences in the mean age, patient selection, and study duration. The mean age of our study was 60 years, with no patients under 40 years old. Considering the relatively higher prevalence of elderly patients within our study, advanced age served as a predictive feature of surgical failure due to poorer conjunctival condition. Furthermore, the patient population in Bowden’s study comprised a significant proportion of younger patients with accompanying secondary glaucoma such as NVG or complex glaucoma. However, our study showed less of such a trend, indicating instead that older age groups tended to have a higher incidence of secondary glaucoma like NVG. This difference in findings compared to previous study could be attributed to patient selection bias. Additionally, the short-term period of 1 year is a contributing factor to the likelihood of surgical failure. In previous study, the increase in surgical failure with younger age was attributed to the longer life expectancy of younger patients, which resulted in a higher probability of reoperation during long-term follow up. However, within the short term follow up of one year, such bias can be reduced, as observed in our study.

Another significant feature was the position of the Ahmed valve, which is commonly significantly associated with surgical failure in the majority of combined dataset models. Our study revealed that the Ahmed valve insertion in the ciliary sulcus is associated with an increased risk of surgical failure. Notably, in this study, sulcus insertion was commonly performed in a specific subset of patients with pseudophakia and specific types of glaucoma with a low success rate, including closed-angle glaucoma, uveitic glaucoma, and NVG. Therefore, additional thorough research is needed to confirm the result. However, this finding serves as correctable factor when planning Ahmed valve implantation, as previous studies have not found differences other than the impact of positioning on corneal epithelial cells [36]. Another correctable risk factor during surgery was identified in this study. When analyzing the combined dataset A + B + C, the type of patch graft showed a high feature importance in influencing surgical failure. Specifically, the relative use of bovine pericardial patch appeared to be a risk factor for surgical failure compared to the use of human donor sclera patch. Even though the evidence to explain this is somewhat lacking and it was not a significant result in other dataset combinations, it is worth noting as a correctable factor during surgery and a new finding that has not been compared in previous studies.

While not commonly confirmed and with somewhat lower feature importance, we identified interesting results in a data subset. First, the use of benzodiazepine was observed as the only associated factor among psychiatric medications, considering the combination of datasets A and C which include psychiatric medication information. A previous study has reported that the use of benzodiazepine is significantly associated with the risk of angle closure glaucoma [37]. Further investigation is required to identify the pathophysiology between benzodiazepine and the surgical failure of Ahmed valve implantation. However, confirming the use of benzodiazepine and modifying it preoperatively is associated with surgical success. Second, the treatment compliance of anti-hypertensive drugs and statins is the only systemic risk factors associated with surgical failure when comparing the results from the combination of datasets that include systemic information. Our study did not categorize the mechanisms of various antihypertensive medications that patients were taking, thus determining which mechanism might have influenced the success rate postoperatively is impossible. However, the possibility that patients taking antihypertensive drugs may experience a lower surgical success rate indicates a higher likelihood of hypertension in those who underwent surgical failure than in those receiving no medication. The finding of our study is explainable, considering the mechanism through which high blood pressure can influence IOP [38].

Our surgical outcome studies, which have applied the ML model, have demonstrated similar AUROC compared to previous studies in ophthalmology. Gleichgerrcht et al. predicted temporal lobe epilepsy surgical outcomes with an AUROC 0.88 using the ML mechanism [39]. Hollon et al. used an ML algorithm to predict early outcomes of pituitary adenoma surgery and found the effective performance of ML with AUROC of 0.827 using LR with Elastic Net [40]. Our optimized XGBoost model outperformed LR and SVR model using AUROC as evaluation metrics, and showed an AUROC of 0.801 using the dataset A, B, and C. The results of our study are as significant as the initial research utilizing in the Ahmed valve implantation because of the comparable AUROC values. Although the current model has lower accuracy values compared to AUROC, we considered this a reasonable outcome given the complexity of the physiology and the small sample size. As the program continues to improve, it is expected to demonstrate more effective performance in surgical procedures, similar to the findings reported by X song el al., who observed good performance of their decision model for ptosis surgery [41].

Our study revealed that machine performance constructed by combining dataset B and C was the highest when considering AUROC alone. However, the model created by combining only dataset B or C showed better or similar performance than the model that combined all datasets, considering ACC and F1 score. These results can be attributed to the result reported in the previous study, indicating that the performance of machine tends to deteriorate when the number of variables exceeds the number of patients [42]. The study concluded that the efficiency of developing predictive models decreases when the number of predictors exceeds the number of participants. The observed outcome can be attributed to the limited number of participants and the large number of variables involved, considering the demographics of our study, which involved 45 patients and 31 variables in the internal validation. However, the performance of the combining model was superior compared to the models created only with a single dataset. In conclusion, our study results revealed that incorporating systemic or psychiatric factors, in addition to ophthalmologic factors, when constructing a predictive model, significantly helps predict surgical failure although determining whether combining all variables would result in improved performance is impossible. This finding should also be considered in the further studies.

This study has several limitations. First is its retrospective nature and the relatively small sample size, which includes patients who have undergone previous glaucoma surgery. Studies using ML revealed that the performance of the model tends to improve when including a larger number of patients. Furthermore, despite the diverse severity of patients’ glaucoma, we were unable to find suitable test results for preoperative evaluation. Further studies should incorporate a prospective design to include a larger number of patients, categorizing the severity of glaucoma. Second, the 1-year postoperative follow-up period is insufficient for predicting surgical outcomes of Ahmed valve implantation. Although the follow-up period might be insufficient, maintaining favorable outcomes during the first 1-year is important for long-term IOP management after the Ahmed valve implantation. Therefore, our study provides information about the significant factors that require attention during the first 1-year period postoperatively to improve long-term surgical outcomes of the Ahmed valve implantation. Third, studies using ML techniques needs to leverage big data from multi-center to overcome the limitations of data. However, our study was limited to using data from a single tertiary hospital, which compromised its representativeness. Future studies need to be designed as multicenter to enhance generalizability of the findings. Fourth, the data were imbalanced with a low incidence of outcome. Despite the low rate of outcomes is a real-world setting, there is significance in this study being the first attempt to utilize ML techniques for Ahmed valve surgery. Fifth, we developed a predictive model using extracted features from the medical image rather than using the image itself. We utilized multiple data sources, but all of them were structured data. Several recent studies have reported the use of eye images themselves to develop predictive models or improve their performance [43–45]. Therefore, further research is needed to utilize the images themselves when developing data-integrated predictive models.

## Conclusions

In conclusion, we have revealed that the use of ML offers some predictive value in predicting the outcomes of Ahmed valve implantation at 1 year. Furthermore, incorporating not only ophthalmic factors but also systemic and psychiatric factors as additional variables improves model performance. Despite some limitations, we believe that our study will serve as an important step in applying ML not only for diagnosing glaucoma but also for incorporating it into the expanding range of surgical techniques for patients with glaucoma. Such an approach would aid in individually predicting surgical outcomes and selecting appropriate surgical techniques. Hence, further studies that use multicenter designs and larger patients are necessary to improve model performance.

### Electronic supplementary material

Below is the link to the electronic supplementary material.

Supplementary Material 1

Supplementary Material 2: Peer review

## Data Availability

No datasets were generated or analysed during the current study.

## Declarations

### Peer review

The peer review reports can be found at 10.1186/s12886-024-03510-w.

## Abbreviations

ABC: Ahmed Baerveldt Comparison Study
AUROC: Area under the receiver operating characteristics curve
AVB: Ahmed Versus Baerveldt Study
BSS: Balanced salt solution
GDI: Glaucoma drainage device implantation
IOP: Intraocular pressure
LR: Logistic regression
ML: Machine learning
NVG: Neovascular glaucoma
SVM: Support vector machines
XGBoost: Extreme gradient boosting
