# Supplementary material for: Evaluation of machine learning approach for surgical results of Ahmed valve implantation in patients with glaucoma
Source: BMC Ophthalmol. 2024 Jun 11;24:248. doi: 10.1186/s12886-024-03510-w (PMC11167936; doi:10.1186/s12886-024-03510-w)
Supplement: Supplementary file 1 — Supplementary Material 1 [file 12886_2024_3510_MOESM1_ESM.docx]

Supplementary Table 1. Additional performance results of predictions models

| **Datasets** | **A + B** | | | **A + C** | | | **A + B + C** | | |
| --- | --- | --- | --- | --- | --- | --- | --- | --- | --- |
| **Performance metrics** | **SVM** | **LR** | **XGB** | **SVM** | **LR** | **XGB** | **SVM** | **LR** | **XGB** |
| ACC | 0.64 | 0.27 | 0.84 | 0.67 | 0.53 | 0.69 | 0.47 | 0.31 | 0.64 |
| AUROC | 0.61 | 0.47 | 0.78 | 0.51 | 0.65 | 0.76 | 0.58 | 0.50 | 0.80 |
| AURPC | 0.18 | 0.14 | 0.09 | 0.16 | 0.19 | 0.10 | 0.17 | 0.14 | 0.09 |
| F1 score | 0.39 | 0.30 | 0.59 | 0.35 | 0.40 | 0.50 | 0.33 | 0.31 | 0.47 |
| Sensitivity | 0.71 | 1.00 | 0.71 | 0.57 | 1.00 | 1.00 | 0.86 | 1.00 | 1.00 |
| Specificity | 0.63 | 0.13 | 0.87 | 0.68 | 0.45 | 0.63 | 0.39 | 0.31 | 0.58 |
| Precision | 0.26 | 0.18 | 0.50 | 0.25 | 0.25 | 0.33 | 0.21 | 0.18 | 0.30 |

ACC = accuracy; AUROC = area under the receiver operating characteristics curve; AUPRC = area under the precision recall curve; SVM = support vector machine, LR = logistic regression; XGB = extreme gradient boosting

Supplementary Table 2. The positive or negative contribution to the prediction of surgical failure

| **Rank** | **Dataset A** | **Dataset A + B** | **Dataset A + C** | **Dataset A + B + C** |
| --- | --- | --- | --- | --- |
| **1** | Preoperative corneal central thickness (CCT) | Age | Age | Age |
| **2** | Valve in sulcus during operation | Valve in sulcus during operation | Preoperative corneal central thickness (CCT) | Valve in sulcus during operation |
| **3** | Preoperative alpha agonist use | Preoperative topical medication use | Valve in sulcus during operation | Used pericardial  patch during  operation |
| **4** | Used pericardial patch during operation | Preoperative intraocular pressure (IOP) | Preoperative intraocular pressure (IOP) | Statin use before operation |
| **5** | Age | Preoperative oral carbonic anhydrase inhibitor (CAI) use | Preoperative topical medication use | POAG diagnosis |
| **6** |  | Anti-hypertensive medications before operation | POAG diagnosis | Preoperative topical medication use |
| **7** |  | Pseudophakia | Preoperative alpha agonist use |  |
| **8** |  |  | Female |  |
| **9** |  |  | Benzodiazepine use before operation |  |

The important predictive features of the XGBoost model from four types of dataset combinations were ranked. Red represents the positive contributors to surgical failure prediction, while blue indicates the negative contributors. POAG = primary open angle glaucoma
