# Supplementary material for: Evaluation of machine learning approach for surgical results of Ahmed valve implantation in patients with glaucoma
Source: BMC Ophthalmol. 2024 Jun 11;24:248. doi: 10.1186/s12886-024-03510-w (PMC11167936; doi:10.1186/s12886-024-03510-w)
Supplement: Supplementary file 2 — Supplementary Material 2: Peer review [file 12886_2024_3510_MOESM2_ESM.zip › Point-by-point response V1.pdf]

Response Letter (Submission ID: 38a92ed5-f8ac-4e02-9c88-67d0f2833b09)

Dear Editor and Reviewers,

We are grateful to the editor and reviewers for the helpful comments. We have responded to the editor's and reviewer's comments in a point-by-point manner and revised the manuscript accordingly. We hope that our paper is now acceptable for publication in the ***BMC Ophthalmology***.

Sincerely,

Jaehong Ahn, MD
Department of Ophthalmology, Ajou University School of Medicine
206, Worldcup-ro, Yeongtong-gu, Suwon-si, Gyeonggi-do, 16499, Republic of Korea
Tel: +82-31-219-5255, Fax: +82-31-219-5259, E-mail: chriseye68@gmail.com

REVIEWER COMMENTS

*All line number is based on the clean copy (no tracking version).
*We've added all the references in the response to the end of this document.

Reviewer #1

General Comment:

The authors utilized machine learning algorithms to identify factors that predicted failure in Ahmed valve implantation. I think this is a novel approach to evaluate a relevant clinical question. However, I think there is a need to contrast their findings more with current literature that do not utilize machine learning.

Response:

Thank you for your summary and kind comment. We have responded to your comments in a point-by-point manner and revised the manuscript accordingly.

Comment 1:

Page 7, line 1: Can the author explain their rationale for using a 7:3 data split instead of the usual 8:2?

Response:

We apologize for the insufficient explanation. As reviewer's comment, many studies have been reported using an 8:2 data split. However, it's not necessarily an 8:2 ratio, with studies reporting 75:25 or 7:3 ratios *(Ref. 1-2)*. In the field of ophthalmology, a recent machine learning paper published in JAMA ophthalmology also used a 7:3 data split *(Ref. 3)*. Given this situation, we believe that it may be possible to use a 7:3 data split in the study. Also, in terms of the sample size, we only have 22 outcome samples, so an 8:2 split would result in only 4 outcomes in the test set. Considering this, we believe that 7:3 is more appropriate.

Comment 2:

Page 7, line 12-13: I don't understand this. Are the authors trying to say the ML algorithm that achieved the highest AUROC was selected as the final algorithm? Perhaps, "as" should be used in place of "for"?

Response:

I apologize for the confusion. As you suggested, we have chosen the model with the highest AUROC value as the final algorithm. I have made adjustment to address any awkwardness in expression. Thank you for your kindness. *(Page 8 line 4-5)*

Comment 3:

Page 7, line 21: Are medication data (e.g., psychiatric medication) self-reported or based on hospital EMR?

Response:

Thank you for your question. Our medication data all based on hospital EMR. The information was obtained retrospectively through the examination of patients' medical records. I have added the relevant content to the manuscript. *(Page 8 line 9-10)*

Comment 4:

Were there subjects with history of multiple surgeries? Multiple surgeries may increase

the risk of complications and failure.

Response:

Yes, that's correct. We cannot completely rule out the possibility of an increased risk associated surgical failure, and I have added this aspect as a limitation in the study. *(Page 16 line 19)* However, it was noted that there were no significant difference associated previous surgery history between the surgical success and failure groups. Furthemore, in our study, we analyzed whether a history of multiple surgeries (ex. history of trabeculectomy) could indeed act as a risk factor for surgical failure. However, no consistent findings were observed across most dataset combinations. This suggests that a history of multiple surgeries may not have a significant impact on the outcomes of Ahmed valve insertion surgery over the course of one year, statistically. Of course, it seems that more insightful study should be conducted on this matter. I am very grateful for your insightful observations and questions.

Comment 5:

Any HVF data or classification on the severity of glaucoma?

Response:

Thank you for your question. We have made efforts to categorize the severity of glaucoma due to the potential impacts on the research results, as you suggested. However, as you are aware, defining the severity of glaucoma is somewhat ambiguous. Furthermore, while conducting pre- and post-operative visual field tests, which we consider to be the most meaningful indicator, could help categorize the severity of glaucoma, many patients in our study underwent surgery urgently due to sudden IOP spikes before pre-operative visual field tests could be conducted. Additionally, a significant portion of patients, due to age or low vision, could not undergo the post-operative visual field tests. As a result, many patients encountered difficulties in classifying the severity of glaucoma, which unfortunately was not addressed in our study. We will make sure to be more mindful in patient selection for further studies to address these issues. I will additionally document this aspect as a limitation of the study. Thank you for your insightful comments. *(Page 16 line 21 – 24)*

Comment 6:

Any data on anterior chamber dimension, such as anterior chamber depth, and presence of synechiae?

Response:

I appreciate your pointing out these issues, and I apologize for the oversight in the study. We classified patients based on gonioscopy to differentiate between open angle and closed angle glaucoma, so we did not measure anterior chamber depth separately. Additionally, we did not consider how this aspect might impact the results of the study. While it may be difficult to mention examinations related to anterior chamber dimension for patients, post-synechiae were observed in a total of 8 patients: 3 were diagnosed with uveitic glaucoma, and 5 were diagnosed with neovascular glaucoma. Thank you for comments.

Comment 7:

Page 8, line 17: "included 133 of the 272..." – why only half of the data was used?

Response:

I apologize for any lack of clarity in expression. Out of a total of 272 patients who underwent Ahmed valve implantation, only 133 were included in this study. The other 139

patients were either unable to undergo follow-up observation for 1 year after surgery or had persistently measured visual acuity at hand motion or below both before and after surgery, so excluding them from this study, I will make the adjustment to this content in the manuscript. *(Page 9 line 11-14)*

Comment 8:

Page 9, line 11: "while 7 were among the 45 patients." – Are the authors trying to say 7 out of 45 patients included in the test group had well controlled IOP after implantation?

Response:

I greatly appreciate your meticulous feedback. The content I intended to convey aligns with your remarks, but it seems the expression may have been somewhat awkward. I have revised it accordingly. Thank you for your comments. *(Page 10 line 6-7)*

Comment 9:

Table 2: Can the authors also present the sensitivity/ specificity, and precision/ recall metrics in addition to accuracy and F1?

Response:

We appreciate helpful comment. We added metrics to the table 2 as attached below. *(Page 10 – Page 11)*

Table 2. Performance results of predictions models.

**Datasets**	**A**			**A + B**	**A + C**	**A + B + C**
**Performance metrics**	**SVM**	**Logistic regression**	**XGBoost**	**XGBoost**	**XGBoost**	**XGBoost**
ACC	0.600	0.511	0.756	0.844	0.689	0.644
AUROC	0.598	0.624	0.684	0.782	0.763	0.801
AUPRC	0.172	0.184	0.108	0.095	0.100	0.095
F1 score	0.400	0.389	0.421	0.588	0.500	0.467
Sensitivity	0.857	1.000	0.571	0.714	1.000	1.000
Specificity	0.553	0.421	0.789	0.868	0.632	0.579
Precision	0.261	0.241	0.333	0.500	0.333	0.304

Comment 10:

Table 2: The highest AUROC achieved was 0.801 when applying XGBoost on dataset A+B+C. However, this AUROC is accompanied with the lowest accuracy and F1 score. Can the authors comment on this?

Response:

Thank you for your comment. This is due to differences in the nature of AUROC and other metrics. While AUROC includes performance over a range of thresholds, accuracy and F1 score are based on performance at a single threshold where Youden's index is at its maximum. Imbalances in the data can cause a discrepancy between the AUROC and performances including accuracy and F1 score at a particular point in time *(Ref 4).* A high AUROC means that the model is able to distinguish between positive and negative classes

well at the overall threshold. However, the optimal threshold set by Youden's Index may not fully reflect the imbalance in the dataset. For example, at this threshold, if the model does not predict the minority classes well and overpredicts the majority classes, the accuracy and F1 Score may appear low. Although this data imbalance is a common problem in medicine, it is a limitation of our study, and we have added the following to the limitations. *(Page 17 line 8-11)*

Comment 11:

Table 2: Given that the dataset is highly unbalanced, the AUPRC and F1 score should be more appropriate but the AUPRC is around 0.10. Can the authors comment and reconcile on this?

Response:

We appreciate the reviewer’s comment. We agree with the reviewer’s comment that the AUPRC and F1 score should be more appropriate when the dataset is highly unbalanced. However, the imbalance in the dataset also causes low AUPRC and F1 scores. In our group's study of predictive models using the same algorithm *(Ref 5),* the unbalanced dataset resulted in an AUPRC of 0.148, which is similar to this study. In that study, we found that the AUPRC improved from 0.148 to 0.855 when analyzed after correcting for the imbalance in the dataset. To summarize, AUPRC is important when unbalanced, but on the other hand, AUPRC can be low due to unbalance. The previous study used a large dataset of 200,000 patients, which allowed for further analysis to correct for imbalances in the dataset, but we were unable to perform a balancing analysis with 150 patients.

Comment 12:

Discussion: Which set of results do the authors propose the readers to focus on? The use of XGBoost on dataset A, A+B, A+C, or A+B+C (considering the drop in accuracy as AUROC increases from A per-se to A+B+C)?

Response:

I am grateful for your insightful comments and advice. We indeed deliberated extensively on this aspect when beginning our research. Typically, accuracy is used for balanced datasets, i.e., when the classes are equally distributed, while AUROC is known to be well-suited for imbalanced datasets. Therefore, we aimed for AUROC values to be the focus, and consequently, the results from the A+B+C combination dataset, which had the highest AUROC value (0.801), are deemed significant for readers. The discussion was also based on this premise. Thank you very much for your question.

Comment 13:

Page 11, line 11: I assume this is increasing age? If so, how does this compare to Bowden’s observation that younger age is associated with surgical failure?

Response:

We have ultimately concluded that increasing age is a factor associated with surgical failure. This aspect is also mentioned in the paragraph starting from the 16th line on page 13, as referenced in your last comment. We will further address and supplement this in the final remarks by providing additional responses and content. *(Page 13 line 15- Page 14 line 4)*

Comment 14:

Page 11, line 24: "Previously published studies frequently had limitations in their retrospective design." – but this is also a retrospective study.

Response:

I have revised the content as you pointed out. The intended meaning was that, since these studies were retrospective, the identified risk factors are difficult to modify before surgery. The previous wording did not convey this intention accurately. I have corrected this part. Thank you. <u>*(Page 13 line 2-4)*</u>

Comment 15:

Page 12, line 1-2: Can the authors explain more on the modifiable risk factors identified in their study?

Response:

Preoperative oral medications or the type of antiglaucoma medication used prior to surgery could be considered as modifiable risk factors. In our study, when analyzing the combination of datasets A+B+C, the usage of statins showed relatively significant feature importance, suggesting its meaningful impact. Additionally, factors such as the type of patch used during surgery and the positioning of the tube (anterior chamber vs. sulcus) could also be modifiable risk factors during surgery. <u>*(Page 13 line 3-11)*</u>

Comment 16:

Page 12, line 6-21: This info has been and should be reported elsewhere in the manuscript.

Response:

Yes, I will condense the previously mentioned content and integrate it into another paragraph for clarity. Thank you for your advice.

Comment 17:

Page 13, line 3-6: Instead of comparing to application in other diseases, I would prefer to see the authors discuss and compare/contrast their results with reference to current literature that did not utilize ML to assess for factors for Admed valve implantation failure. E.g., did the ML reported something previously unknown or in contrast to current literature?

Response:

Thank you for the helpful advice. While the content you mentioned was intended to derive significance from comparing AUROC values with other studies, it seems to have some awkwardness. I have rephrased and organized the content, summarizing it and incorporating related information. Thank you for your kindness. <u>*(Page 15 line 14 – 18)*</u>

Comment 18:

Page 13, line 8: "relatively high AUROC" - But this came with a lower accuracy. Need to balance this point.

Response:

We appreciate helpful comment. As mentioned by the reviewer, we've made the following changes to address the issue. <u>*(Page 15 line 22 – Page 16 line 1)*</u>

Comment 19:

Page 13, line 16-20: Although the subjects age may differ, the direction of association should generally be the same? Can the authors explain this contrasting finding in more detail? Also, how does the shorter study duration explains the discrepancies. A shorter time period will likely lead to less cases of surgical findings but how does this leads to a reserve in observation that increased age increases surgical failure as compared to younger age previously reported?

Response:

In previous studies, two main reasons were mentioned for the increase in surgical failure with younger age. First, it was attributed to differences in patient populations. In the Bowden's study, it was often observed that younger patients had a higher prevalence of difficult-to-control IOP after surgery, particularly in cases of secondary glaucoma such as neovascular glaucoma (NVG) or complex glaucoma. However, our study showed less of such a trend, indicating instead that older age groups tended to have a higher incidence of secondary glaucoma like NVG. This difference in findings compared to previous studies could be attributed to patient selection bias. Secondly, differences in follow-up periods were considered. In the Bowden's study, the increase in surgical failure with younger age was thought to be due to the longer life expectancy of younger patients, leading to a higher probability of reoperation from a long-term perspective. This perspective is related to the tendency for Ahmed valve implantation surgery to show decreasing success rates over time. However, in our study, focusing on short-term outcomes over one year reduces the impact of such bias. In other words, within the short timeframe of 1 year, older patients may appear to have a lower short-term success rate due to the advancement of glaucoma severity or poorer conjunctival conditions, which are more common in older age groups. This could be another factor contributing to the difference in results. We have also included some additional explanation of these points in the main text. Thank you for your comments. *(Page 13 line 12 – Page 14 line 4)*

Reviewer #2

General Comment:

In this manuscript, the authors investigate the use of machine learning to predict outcomes of Ahmed valve implantation in glaucoma patients at Ajou University Hospital from 2017 to 2021. It analyzes retrospective data, considering patients' demographics, health, and eye parameters. On this basis, the study also identifies predictors of surgical success or failure. This work makes an important step in applying ML not only for diagnosing glaucoma but also for incorporating it into the expanding range of surgical techniques for patients with glaucoma. The manuscript is well-organized and clearly stated. I would suggest accepting it after the following minor concerns are addressed:

Response:

Thank you for participating as a reviewer. We believe that the results of this study provide necessary evidence in the clinical field. We have responded to your comments in a point-by-point manner and revised the manuscript accordingly.

Comment 1:

The background section mentions numerous studies that utilize machine learning models for predicting surgical outcomes, but the specific goals and benefits of employing these models are not clearly defined. Please clearly clarify the objectives and advantages of using machine learning in this context.

Response:

Thank you for your valuable advice. I have incorporated the points you mentioned into the background section. I appreciate it. *(Page 4 line 19 -22)*

Comment 2:

In the background section, additional information regarding the Ahmed valve would be beneficial. Please give more background information about Ahmed valve.

Response:

Thank you for your valuable advice. I have recorded additional information based on your suggestions. Thank you. *(Page 3 line 12-16)*

Comment 3:

In the methods section: “This study included cases with previous cataract surgery, vitrectomy, and trabeculectomy”. Do these previous surgeries impact the outcomes of the current surgery, and could they be considered as independent covariates in your statistical analysis?

Response:

We appreciate the reviewer’s comment. We agree with the reviewer’s comment that previous surgeries could impact the outcomes of the current surgery. We included the presence of previous surgery as a predictor in our predictive model. As a predictor in a predictive model is the same as an independent variable, we considered surgical history as an independent variable in our model. However, contrary to our expectations, it was not included in the top predictors because it had less impact than the other variables.

Comment 4:

In the methods section : "…any fixed-dose combination drugs were considered as two separate drugs". Please explain the rationale behind this decision?

Response:

I have thoroughly understood the points you raised. While it may be difficult to provide a clear rationale, I have determined that it is appropriate to consider the two drugs separately for statistical analysis because they have different mechanisms of action, particularly in the case of glaucoma eye drops. As mentioned in the *Ref 6* below, the paper describes that when analyzing fixed combination drugs, it is appropriate to consider them as two separate drugs for analysis. Therefore, in this paper, we also conducted statistical analysis by considering the fixed combination drug as two separate drugs. Furthermore, as in the following paper renowned for Tube shunt surgery *(Ref 7)*, the number of eye drops was also checked in the same manner as in this paper. *(Page 6 line 5)*

Comment 5:

In the methods section, it would be helpful to know which software was utilized for the machine learning simulations. Please describe what software is used for machine learning simulation

Response:

We appreciate the thoughtful comment. As reviewer's comment, we have added the following to the methods. *(Page 8 line 23 – Page 9 line 1)*

Comment 6:

In the methods section, a more detailed explanation of the XGBoost algorithm in the methods section would enhance the reader's understanding of why and how it was chosen for this study. Please provide a more detailed explanation for XGBoost.

Response:

Thank you for your advice. As reviewer's comment, we have added the following to the methods. *(Page 7 line 17 – Page 8 line 1*)

Comment 7:

In the methods section, the initial exclusive use of Dataset A to assess the performance of the three classifiers raises questions. If a different dataset were employed, might the outcomes change? Furthermore, after combining datasets (Dataset A+B, Dataset A+C, Dataset A+B+C), could other classifiers such as SVM or LR potentially offer improved results?

Response: Thank you for your comment. As reviewer's comment, model development and performance evaluation using SVM and LR were also performed on the combined data (A+B, A+C, A+B+C). As a result, XGBoost performed the best in all other datasets, so it seems that the existing method is appropriate. Other classifiers also showed performance improvements on combined data, but not in all cases. For SVM, performance improved when medical data was added, and for logistic regression, performance improved when psychiatric data was added. The overall result is shown in the table below. We've added the table below to the supplement and added an additional sentence to the results section.

Supplementary Table 2. Additional performance results of predictions models

Datasets	A + B			A + C			A + B + C		
Performance metrics	SVM	LR	XGB	SVM	LR	XGB	SVM	LR	XGB
ACC	0.64	0.27	0.84	0.67	0.53	0.69	0.47	0.31	0.64
AUROC	0.61	0.47	0.78	0.51	0.65	0.76	0.58	0.50	0.80
AURPC	0.18	0.14	0.09	0.16	0.19	0.10	0.17	0.14	0.09
F1 score	0.39	0.30	0.59	0.35	0.40	0.50	0.33	0.31	0.47
Sensitivity	0.71	1.00	0.71	0.57	1.00	1.00	0.86	1.00	1.00
Specificity	0.63	0.13	0.87	0.68	0.45	0.63	0.39	0.31	0.58
Precision	0.26	0.18	0.50	0.25	0.25	0.33	0.21	0.18	0.30

Comment 8:

The color similarity between the LR and SVM curves in Figure 2A, and between Dataset A+B and Dataset A+C in Figure 2B, poses a challenge for distinguishing them.

Response: We appreciate helpful comment. As mentioned by the reviewer, we've revised the figure as attached below.

Figure 2.

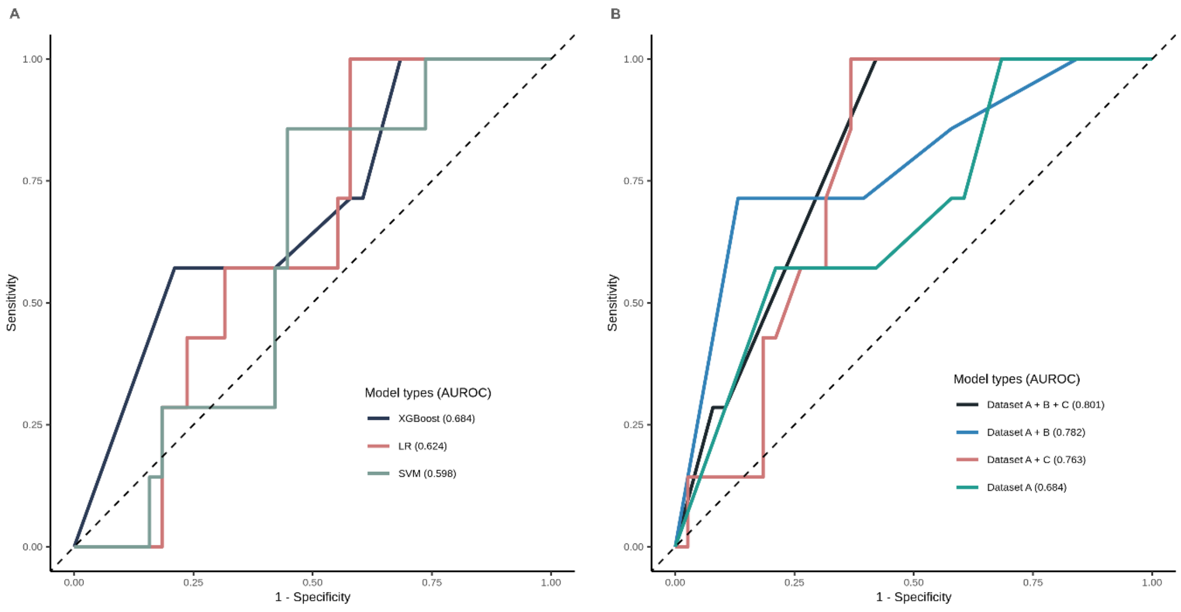

Comment 9:

In the first paragraph of the discussion: "...and the high performance of our models confirms that the ML algorithms using preoperative patient data would enable clinicians to provide tailored treatment options." Please provide further details on "tailored treatment options".

Response:

Thank you for the valuable advice. The authors predict that in patients with relatively higher preoperative risk factors, the success rate of Ahmed valve implantation may decrease. In such cases, we believe that choosing alternative surgical methods before surgery or conducting short-term follow-up after surgery while initiating early use of intraocular pressure-lowering medications can enable more individualized approaches to IOP management and glaucoma prognosis management. We also considered that this study, while modest, could serve as a foundation for such research. Accordingly, we have added some additional content to the manuscript. Thank you. <u>(Page 12- line 13-14)</u>

Comment 10:

In the second paragraph of the discussion : "Moreover, most of the identified risk factors in these studies are difficult to modify or influence preoperatively." Given the cited limitation in other studies, how does this study address or improve upon the limitation? A comprehensive explanation would be appreciated.

Response:

I greatly appreciate your insightful and valid points. I have incorporated your suggestions into the text. <u>*(Page 13 – line 2-7)*</u>

Comment 11:

It would be beneficial to include the reference "A clinical decision model based on machine learning for ptosis" in the third paragraph of the discussion to support your argument.

Response:

I am very grateful for your advice. I have incorporated your suggestions and further refined the content for completion. Thank you. <u>*(Page 15 line 25 – Page 16 line 1)*</u>

Comment 12:

In the fifth paragraph of the discussion: "Additionally, the short-term period of 1 year is a contributing factor to the likelihood of surgical failure". Could you clarify the basis for this assertion?

Response:

Thank you for your kind words. Following your advice, I have added further explanation to this section. <u>*(Page 13 line 26 – Page 14 line 4)*</u>

Comment 13:

The article critiques the retrospective nature of previous studies, yet this research also employs a retrospective design. Could this be considered a contradiction?

Response:

I have revised the content as you pointed out. The intended meaning was that, since these studies were retrospective, the identified risk factors are difficult to modify before surgery. The previous wording did not convey this intention accurately. I have corrected this part. Thank you. (*Page 13 line 2-9)*

Comment 14:

To facilitates performance enhancement of multi-classification models for medical images, these papers could be referred to:
(1) Sahoo, M., Ghorai, S., Pal, S. and Mitra, M., 2022. A Multi-Layer stacked ensemble classifier model for improved classification accuracy of Maculopathy gradation. Displays, 73, p.102213.
(2)Zhu X, Song X, Min X, Zhou H, Sun W, Wang J, Zhai G. Calculation of ophthalmic diagnostic parameters on a single eye image based on deep neural network. Multimed Tools Appl 2022;81:2311-2331
(3)Liu W, Zhou L, Yang X. High-order features of a single linear corneal laceration image are valuable biomarkers in an intelligent multimodal analytic strategy for corneal laceration reconstruction[J]. Displays, 2023, 79: 102507.

Response:

Thank you for your kind comment. In our study, we developed a predictive model using extracted features from the medical image rather than using the image itself. We utilized multiple data sources, but all of them were structured data. As the reviewer commented, we believe that using the medical image itself is the way forward for future model development using multiple data sources. We have added the following to the limitations and added the suggested paper to the references. *(Page 17 line 11-15)*

Reference

1. Lee DY, Kim C, Lee S, et al. Psychosis relapse prediction leveraging electronic health records data and natural language processing enrichment methods. *Frontiers in Psychiatry*. 2022;13

2. An C, Park YW, Ahn SS, Han K, Kim H, Lee S-K. Radiomics machine learning study with a small sample size: Single random training-test set split may lead to unreliable results. *PLoS One*. 2021;16(8):e0256152.

3. Wang Y, Du R, Xie S, et al. Machine Learning Models for Predicting Long-Term Visual Acuity in Highly Myopic Eyes. *JAMA ophthalmology*. 2023;141(12):1117-1124.

4. Bekkar, M., Djemaa, H.K. and Alitouche, T.A., 2013. Evaluation measures for models assessment over imbalanced data sets. J Inf Eng Appl, 3(10).

5. Lee DY, Oh AR, Park J, et al. Machine learning-based prediction model for postoperative delirium in non-cardiac surgery. *BMC psychiatry*. 2023;23(1):317.

6. Inoue, K., Shiokawa, M., Kunimatsu-Sanuki, S., Tomita, G., & Ishida, K. (2024). Switching to Brimonidine/Ripasudil Fixed Combination from Brimonidine + Ripasudil. Clinical Ophthalmology, 18, 423–430.

7. GEDDE, Steven J., et al. Treatment outcomes in the primary tube versus trabeculectomy study after 3 years of follow-up. Ophthalmology, 2020, 127.3: 333-345.
