# Supplementary material for: Evaluation of machine learning approach for surgical results of Ahmed valve implantation in patients with glaucoma
Source: BMC Ophthalmol. 2024 Jun 11;24:248. doi: 10.1186/s12886-024-03510-w (PMC11167936; doi:10.1186/s12886-024-03510-w)
Supplement: Supplementary file 2 — Supplementary Material 2: Peer review [file 12886_2024_3510_MOESM2_ESM.zip › Reviewer Xuefei Song report V1.docx]

**Reviewer 2: Xuefei Song**

Submitted: **02 Apr 2024**

#### **Feedback for the author(s)**

In this manuscript, the authors investigate the use of machine learning to predict outcomes of Ahmed valve implantation in glaucoma patients at Ajou University Hospital from 2017 to 2021. It analyzes retrospective data, considering patients' demographics, health, and eye parameters. On this basis, the study also identifies predictors of surgical success or failure. This work makes an important step in applying ML not only for diagnosing glaucoma but also for incorporating it into the expanding range of surgical techniques for patients with glaucoma. The manuscript is well-organized and clearly stated. I would suggest accepting it after the following minor concerns are addressed: 1.The background section mentions numerous studies that utilize machine learning models for predicting surgical outcomes, but the specific goals and benefits of employing these models are not clearly defined. Please clearly clarify the objectives and advantages of using machine learning in this context. 2.In the background section, additional information regarding the Ahmed valve would be beneficial. Please give more background information about Ahmed valve. 3.In the methods section: “This study included cases with previous cataract surgery, vitrectomy, and trabeculectomy”. Do these previous surgeries impact the outcomes of the current surgery, and could they be considered as independent covariates in your statistical analysis? 4.In the methods section : “…any fixed-dose combination drugs were considered as two separate drugs”. Please explain the rationale behind this decision? 5.In the methods section , it would be helpful to know which software was utilized for the machine learning simulations. Please describe what software is used for machine learning simulation. 6.In the methods section, a more detailed explanation of the XGBoost algorithm in the methods section would enhance the reader's understanding of why and how it was chosen for this study. Please provide a more detailed explanation for XGBoost. 7.In the methods section, the initial exclusive use of Dataset A to assess the performance of the three classifiers raises questions. If a different dataset were employed, might the outcomes change? Furthermore, after combining datasets (Dataset A+B, Dataset A+C, Dataset A+B+C), could other classifiers such as SVM or LR potentially offer improved results? 8.The color similarity between the LR and SVM curves in Figure 2A, and between Dataset A+B and Dataset A+C in Figure 2B, poses a challenge for distinguishing them. 9.In the first paragraph of the discussion : "...and the high performance of our models confirms that the ML algorithms using preoperative patient data would enable clinicians to provide tailored treatment options." Please provide further details on "tailored treatment options". 10.In the second paragraph of the discussion : "Moreover, most of the identified risk factors in these studies are difficult to modify or influence preoperatively." Given the cited limitation in other studies, how does this study address or improve upon the limitation? A comprehensive explanation would be appreciated. 11.It would be beneficial to include the reference "A clinical decision model based on machine learning for ptosis" in the third paragraph of the discussion to support your argument. 12.In the fifth paragraph of the discussion: "Additionally, the short-term period of 1 year is a contributing factor to the likelihood of surgical failure". Could you clarify the basis for this assertion? 13.The article critiques the retrospective nature of previous studies, yet this research also employs a retrospective design. Could this be considered a contradiction? 14. To facilitates performance enhancement of multi-classification models for medical images, these papers could be referred to: (1) Sahoo, M., Ghorai, S., Pal, S. and Mitra, M., 2022. A Multi-Layer stacked ensemble classifier model for improved classification accuracy of Maculopathy gradation. Displays, 73, p.102213. (2)Zhu X, Song X, Min X, Zhou H, Sun W, Wang J, Zhai G. Calculation of ophthalmic diagnostic parameters on a single eye image based on deep neural network. Multimed Tools Appl 2022;81:2311-2331 (3)Liu W, Zhou L, Yang X. High-order features of a single linear corneal laceration image are valuable biomarkers in an intelligent multimodal analytic strategy for corneal laceration reconstruction[J]. Displays, 2023, 79: 102507.
