# Supplementary material for: Evaluation of machine learning approach for surgical results of Ahmed valve implantation in patients with glaucoma
Source: BMC Ophthalmol. 2024 Jun 11;24:248. doi: 10.1186/s12886-024-03510-w (PMC11167936; doi:10.1186/s12886-024-03510-w)
Supplement: Supplementary file 2 — Supplementary Material 2: Peer review [file 12886_2024_3510_MOESM2_ESM.zip › Reviewer Xuefei Song Report V2.docx]

**Reviewer 1: Xuefei Song**

Submitted: **08 May 2024**

#### **Feedback for the author(s)**

The current version is accepctable for publication.
