# Supplementary material for: Evaluation of machine learning approach for surgical results of Ahmed valve implantation in patients with glaucoma
Source: BMC Ophthalmol. 2024 Jun 11;24:248. doi: 10.1186/s12886-024-03510-w (PMC11167936; doi:10.1186/s12886-024-03510-w)
Supplement: Supplementary file 2 — Supplementary Material 2: Peer review [file 12886_2024_3510_MOESM2_ESM.zip › Reviewer Zhi Da Soh report V1.docx]

**Reviewer 1: Zhi Da Soh**

Submitted: **21 Mar 2024**

**Feedback for the author(s)**

Dear editor,

The authors utilized machine learning algorithms to identify factors that predicted failure in Admed valve implantation. I think this is a novel approach to evaluate a relevant clinical question. However, I think there is a need to contrast their findings more with current literature that do not utilize machine learning. My specific comments are as follows:

Page 7, line 1: Can the author explain their rationale for using a 7:3 data split instead of the usual 8:2?

Page 7, line 12-13: I don’t understand this. Are the authors trying to say the ML algorithm that achieved the highest AUROC was selected as the final algorithm? Perhaps, “as” should be used in place of “for”?

Page 7, line 21: Are medication data (e.g., psychiatric medication) self-reported or based on hospital EMR?

Table 1:

Were there subjects with history of multiple surgeries? Multiple surgeries may increase the risk of complications and failure.

Any HVF data or classification on the severity of glaucoma?

Any data on anterior chamber dimension, such as anterior chamber depth, and presence of synechiae?

Page 8, line 17: “included 133 of the 272...” – why only half of the data was used?

Page 9, line 11: “while 7 were among the 45 patients.” – Are the authors trying to say 7 out of 45 patients included in the test group had well controlled IOP after implantation?

Table 2:

Can the authors also present the sensitivity/ specificity, and precision/ recall metrics in addition to accuracy and F1?

The highest AUROC achieved was 0.801 when applying XGBoost on dataset A+B+C. However, this AUROC is accompanied with the lowest accuracy and F1 score. Can the authors comment on this?

Given that the dataset is highly unbalanced, the AUPRC and F1 score should be more appropriate but the AUPRC is around 0.10. Can the authors comment and reconcile on this?

Discussion: Which set of results do the authors propose the readers to focus on? The use of XGBoost on dataset A, A+B, A+C, or A+B+C (considering the drop in accuracy as AUROC increases from A per-se to A+B+C)?

Page 11, line 11: I assume this is increasing age? If so, how does this compare to Bowden’s observation that younger age is associated with surgical failure?

Page 11, line 24: “Previously published studies frequently had limitations in their retrospective design.” – but this is also a retrospective study.

Page 12, line 1-2: Can the authors explain more on the modifiable risk factors identified in their study?

Page 12, line 6-21: This info has been and should be reported elsewhere in the manuscript.

Page 13, line 3-6: Instead of comparing to application in other diseases, I would prefer to see the authors discuss and compare/contrast their results with reference to current literature that did not utilize ML to assess for factors for Admed valve implantation failure. E.g., did the ML reported something previously unknown or in contrast to current literature?

Page 13, line 8: “relatively high AUROC” - But this came with a lower accuracy. Need to balance this point.

Page 13, line 16-20: Although the subjects age may differ, the direction of association should generally be the same? Can the authors explain this contrasting finding in more detail? Also, how does the shorter study duration explains the discrepancies. A shorter time period will likely lead to less cases of surgical findings but how does this leads to a reserve in observation that increased age increases surgical failure as compared to younger age previously reported?
